# Metabolomic and metagenomic insights into WFBG-mediated regulation of gut microbiota and metabolism in broilers

**DOI:** 10.1128/aem.01890-25

**Published:** 2025-12-08

**Authors:** Yuanfeng Li, Xianglei Fu, Funing Sun, Mintao Dong, Yanting Wang, Yan Wang, Qi Liu

**Affiliations:** 1 College of Agriculture and Biology, Liaocheng University 697434, Liaocheng, China; Universita degli Studi di Napoli Federico II, Portici, Italy

**Keywords:** wet-fermented brewer’s grains, broilers, growth performance, metabolomics, 16S rRNA

## Abstract

The steady state of gut microbiota is a key factor in regulating the growth of broilers. The regulatory role of wet-fermented brewer’s grain (WFBG) in broiler gut development and microbiota is still elusive. In this study, non-targeted metabolomics and 16S rRNA sequencing analysis were used to investigate the effects of WFBG supplementation on serum metabolites and gut microbiota in 42-day-old broilers. Serum metabolomic analysis identified 546 differentially expressed metabolites (DEMs), with GO and KEGG enrichment analyses showing that specific DEMs were enriched in intestinal development-related pathways, including phenylalanine, tyrosine, tryptophan biosynthesis, and alpha-linolenic acid metabolism. 16S rRNA sequencing analysis showed significant intergroup differences in the relative abundances of *Ligilactobacillus*, *Olsenella*, *Erysipelatoclostridium*, and *Blautia* at the genus level in broiler gut microbiota between the control and WFBG groups. Integrative analysis of 16S rRNA sequencing and non-targeted metabolomics demonstrated that bacterial genera, including *Streptococcus* and *Proteus*, were positively correlated with N6,N6-dimethyllysine and quercetin but negatively associated with 18 DEMs, such as 4-methylbenzenesulfonic acid and deoxycholic acid derivatives. Furthermore, we identified potential biomarkers associated with intestinal development induced by 20% WFBG supplementation. Our findings suggest that the maximum recommended inclusion level of WFBG in broiler feed should not exceed 20%. This study provides novel insights into the molecular mechanisms underlying fiber utilization and intestinal maturation in broilers.

**IMPORTANCE:**

This study investigated the regulatory mechanism of wet-fermented brewer's grain (WFBG) on gut development and microbiota in commercial broilers. Through integrated 16S rRNA sequencing and non-targeted metabolomic analysis, the study not only identified differential gut microbiota, serum metabolites, as well as their correlations, but also discovered potential biomarkers associated with intestinal development induced by 20% WFBG and clarified the maximum recommended inclusion level of WFBG (≤20%). This not only filled the gap in the molecular mechanism underlying WFBG-mediated regulation of fiber utilization and intestinal maturation in broilers but also provided a theoretical basis and practical guidance for the resource utilization of agricultural by-products, precision feeding of broilers, and intestinal health monitoring.

## INTRODUCTION

With the escalating global food crisis, utilizing processed non-traditional agricultural and industrial by-products as substitutes for corn-soybean diets in poultry feed has emerged as a critical strategy (1). Fermented brewer’s grain (FBG), rich in proteins, amino acids, and trace elements, represents a premium animal feed resource. Previous studies have demonstrated that dried brewer’s grain (DBG) applied in poultry (2–4) and livestock (5, 6) can regulate balance of gut microbiota (7), enhance antioxidant capacity (8), and improve apparent nutrient digestibility (2, 9), thereby boosting growth performance and reducing feed costs (10, 11). Notably, wet brewer’s grain (WBG), constituting 85% of brewing waste and by-products (12), has been used in livestock and poultry feed due to its high nutritional content of dietary fiber, amino acids, and polyphenols (13). While WBG has been widely applied in ruminants owing to its unique ruminal digestion and metabolic characteristics (14), its application in poultry is still limited. Fermentation has been shown to significantly improve the apparent digestibility of DBG nutrients in laying hens (15) and ducks (4). However, research on the application of wet-fermented brewer’s grain (WFBG) in broilers remains scarce.

FBG, a prevalent by-product of the brewing industry, contains abundant protein, dietary fiber, and polyphenolic compounds. Nevertheless, its high crude fiber content and antinutritional factors (e.g., phytic acid and tannins) impede its efficient incorporation into broiler diets (2). Microbial fermentation can enhance its nutritional value by degrading fiber components, neutralizing antinutrients, and synthesizing bioactive metabolites such as prebiotics and short-chain fatty acids (SCFAs) (16, 17). The broiler cecum serves as a critical niche for gut microbiota colonization, where microbial community structure is intricately linked to host nutrient absorption, intestinal barrier integrity, and immunomodulation (18–20). Serum metabolomics, meanwhile, offers insights into systemic metabolic perturbations and microbiota–host co-metabolism (21, 22). Current studies primarily emphasize the isolated effects of WFBG on broiler growth performance, yet systematic investigations along the “feed–microbiota–host metabolism” axis remain scarce. Specifically, mechanistic insights into how WFBG modulates the cecal microbiome to influence host metabolic networks are lacking. This study aims to evaluate WFBG as a sustainable feed ingredient for broilers. It is hypothesized that high-dose WFBG supplementation will not compromise broiler growth performance, thereby optimizing commercial production costs and productivity. Specifically, this research intends to investigate the effects of WFBG on growth performance, serum metabolomics, and cecal microbiota in white-feathered broilers.

## MATERIALS AND METHODS

### Animals and treatments

A total of 480 21-day-old (397.18 ± 3.39 g) white-feathered broilers (hybrid of Cobb breed cock and Hy-line brown laying hen) were randomly divided into five groups (6 replicates of 16 birds each). The control group received a basal diet (0% WFBG), whereas experimental groups were administered diets containing 5%, 10%, 20%, and 30% WFBG, respectively, for 21 days in summer. All birds were housed in wire cages measuring 70 cm × 80 cm × 40 cm. The birds were procured from Aoxiang Poultry Industry Co., Ltd., Liaocheng, China. Throughout the experiment, all birds were provided with *ad libitum* access to feed and clean drinking water. For environmental conditions in the broiler house, ambient temperature was maintained at 33°C–35°C in the first week, thereafter gradually reduced by 2°C–3°C per week until stabilization at 22°C–25°C; relative humidity was controlled at 60%–65% consistently; and the light cycle was maintained at 23 h of light and 1 h of darkness to accommodate the lighting requirements of broilers across different growth stages. The basal diet was sourced from a commercial feed manufacturer, with its formulation and nutritional composition detailed in Table 1. This study was reported in adherence to the ARRIVE guidelines, with all methods conducted in compliance with relevant ethical guidelines and regulatory requirements. WFBG was prepared in the laboratory of Liaocheng University, following the detailed procedure described in Li et al. (23). Notably, the precise fiber composition of this WFBG has been measured and fully reported in the same study (23).

**TABLE 1:** Diet composition (as-fed basis) of white-feathered broilers

Ingredients	Count	Nutrient levels	Count
Corn (%)	63.00	ME (kcal/kg)^*b*^	12.15
Soybean meal (CP 44%) (%)	25.00	Dry matter (%)^*c*^	88.60
Corn gluten meal (%)	6.00	Crude protein (%)^*c*^	18.80
Soybean oil (%)	1.50	Lysine (%)^*b*^	1.05
Calcium monophosphate (%)	1.50	Methionine (%)^*b*^	0.50
Calcium carbonate (%)	1.70	Calcium (%)^*c*^	1.00
Premix^*a*^ (%)	1.00	Available phosphorus (%)^*b*^	0.46
Salt (%)	0.30	–^*d*^	–
Total (%)	100.00	–	–

^*a*^ Provided per kilogram of diet: Cu, 8.0 mg; Fe, 100.0 mg; Zn, 80.0 mg; Mn, 110.0 mg; Se, 0.3 mg; choline chloride, 800.0 mg; vitamin E, 20.0 mg; vitamin D_3_, 3,000 IU; vitamin A, 10,000 IU; folic acid, 1.0 mg; vitamin B_12_ (cobalamin), 0.015 mg; menadione sodium bisulfate, 1.3 mg; thiamin, 2.2 mg; riboflavin, 8.0 mg; nicotinamide, 40.0 mg; calcium pantothenate, 10.0 mg; pyridoxine HCl, 4.0 mg; biotin, 0.04 mg.

^*b*^ Calculated values.

^*c*^ Measured values.

^*d*^ – indicates no data.

### Growth performances

Daily feed intake and weekly body weight (BW) were monitored. Weekly calculations were performed for average daily gain (ADG), average daily feed intake (ADFI), and feed conversion ratio (FCR) across three periods: 22–27 days, 28–34 days, and 35–41 days. Mortality was documented in real time, and the BWs of deceased birds were incorporated to adjust FCR values.

### Sample collection

On day 42 of the experiment, one bird per replicate was randomly selected from both the control and 20% WFBG groups for sampling. Each selected bird was fasted for 12 h, with a BW approximating the mean BW of its respective replicate. Sample collection procedures were as follows: blood samples were first obtained from the wing vein, allowed to stand at room temperature for 2 h, then centrifuged at 5,000 × *g* for 10 min at 4°C to isolate the serum. The resulting serum was stored at −80°C for metabolomic analysis. Following blood collection, birds were euthanized via cervical dislocation. For intestinal morphology analysis, the abdominal cavity was dissected to expose the intestinal tract; 1–2 cm segments were collected from three key intestinal regions: duodenum (≈2 cm from the pylorus), jejunum (mid-section of the jejunum), and ileum (≈2 cm from the cecum). Intestinal contents in the segments were gently rinsed with pre-cooled physiological saline to avoid mucosal damage, and the cleaned tissue segments were immediately placed in 4% paraformaldehyde solution (sufficient to submerge the tissue) for fixation, which preserved tissue structure for subsequent paraffin embedding and hematoxylin-eosin (HE) staining. Cecal contents were immediately harvested using sterile EP tubes, flash-frozen in liquid nitrogen, and stored for subsequent microbiome analysis.

### DNA extraction and library preparation

Cecal samples were obtained from broilers fed with 0% or 20% WFBG; the 20% level was selected for its lower FCR (cutting feed costs via less consumption), while 5% offers no economic benefits in large-scale production (higher FCR, lower inclusion). Cecal DNA was isolated from approximately 150 mg samples via a two-step protocol: mechanical disruption using glass beads followed by purification with a magnetic bead-based genomic DNA extraction kit (AU46111-96; Baitaike Biotech, Beijing, China). The procedure strictly adhered to the manufacturer’s adapted guidelines for fecal specimens. The quality of the extracted DNA was evaluated using a Qubit fluorometer (Thermo Fisher Scientific, Waltham, MA, USA), and purity was evaluated by spectrophotometry (NanoDrop, Thermo Fisher Scientific). Subsequently, the V4 hypervariable region of the 16S rRNA gene was amplified via PCR using universal primers 515F/806R (515F: 5′-GTGYCAGCMGCCGCGGTAA-3′, 806R: 5′- GGACTACHVGGGTWTCTAAT-3′). The amplification was performed on a King Fisher thermal cycler with total DNA as the template. PCR amplification was performed under the following conditions: initial denaturation at 98°C for 30 s, followed by 32 cycles at 98°C for 10 s (denaturation), 54°C for 30 s (annealing), and 72°C for 45 s (extension), with a final extension at 72°C for 10 min. PCR products were purified using AMPure XP beads (Beckman Coulter Genomics, Danvers, MA, USA) and quantified via Qubit fluorometry (Invitrogen, Carlsbad, CA, USA). Quality assessment was performed using an Agilent 2100 Bioanalyzer (Agilent, Santa Clara, CA, USA) and Kapa Biosystems Library Quantification Kits (Kapa Biosciences, Woburn, MA, USA).

### Data processing and 16S rRNA sequencing analysis

Raw demultiplexed sequences were processed using CUTADAPT (v.1.9) to remove primers. Paired-end reads were merged with FLASH (v.1.2.8), followed by quality filtering via fqtrim (v.0.94) to remove low-quality reads (*Q* score <20), short sequences (<100 bp), and those with >5% “N” residues. Clean tags were subjected to chimera removal using Vsearch (v.2.3.4), followed by denoising with DADA2 to generate amplicon sequence variants. Taxonomic annotation was performed using QIIME2’s feature classifier against SILVA and NT-16S databases. Alpha- and beta-diversity metrics were calculated in QIIME2, while relative abundance was determined for bacterial taxa. Differential abundance analysis of genera was conducted using the Wilcoxon test (*P* < 0.05), and linear discriminant analysis effect size (LEfSe, LDA ≥3.0, *P* < 0.05) was performed using the stand-alone LEfSe software. Additional visualizations were generated using R language (v.3.4.4).

### Serum metabolite extraction

#### Serum preparation for metabolomics

Based on the growth performance results, serum samples from the 20% WFBG group were selected for metabolomic analysis. Prior to analysis, samples were ice-thawed and homogenized by vortexing for 10 s. Subsequently, 100 µL of serum samples was mixed with 400 µL of an extraction solution (MeOH:ACN, 1:1 vol/vol) containing deuterated internal standards. The mixture underwent vortexing for 30 s, sonication for 10 min in a 4°C water bath, and a 1 h incubation at −40°C to precipitate proteins. Following centrifugation at 12,000 rpm for 15 min at 4°C, the supernatant was transferred to a new glass vial for ultra-high performance liquid chromatography (UHPLC)-tandem mass spectrometry analysis. To prepare quality control samples, equal volumes of supernatants from all samples were pooled and homogenized.

### Metabolomics data capture

LC-MS/MS analyses of both positive and negative metabolites were conducted using an UHPLC system (Orbitrap Exploris, Thermo Fisher Scientific) interfaced with a UPLC BEH Amide column (Waters ACQUITY, 2.1 mm × 50 mm, 1.7 µm) and an Orbitrap Exploris 120 mass spectrometer (Thermo Fisher Scientific). The mobile phase comprised solvent A (containing 25 mmol/L ammonium acetate and 25 mmol/L ammonium solution, pH = 9.75) and solvent B (acetonitrile). Samples were injected at a volume of 2 µL with the autosampler maintained at 4°C. The mass spectrometer, controlled by Xcalibur software (v.4.4, Thermo Fisher Scientific), operated in information-dependent acquisition mode to acquire full-scan MS spectra. Detailed parameters for the metabolomic analysis are provided in File S1.

### Metabolomic analysis

Raw data were converted to mzXML format using the ProteoWizard software (ProteoWizard Software Foundation, USA), followed by processing with a custom in-house program developed in R and built upon XCMS. Metabolite identification was performed using an R package combined with BiotreeDB (v.3.0) (24). Metabolites with a variable importance in projection (VIP) score of >1 and a *P* value of <0.05 were identified as potential biomarkers.

### Correlation network analysis

To perform correlation analysis, metabolomics data were pre-processed by filtering 20 differentially expressed metabolites (DEMs) identified via random forest feature selection. This approach prioritized metabolites with the highest impact on model performance, streamlining subsequent analyses. For metagenomics data, species-level relative abundance values were used for all groups. Hierarchical all-against-all association analysis was conducted with the following parameters: similarity metric: Spearman’s rank correlation coefficient, FDR control: Benjamini–Hochberg method (α = 0.05), and false negative rate: default setting (0.2) for detecting dense association blocks. Significant associations were compiled into a table, and heat map visualization was performed using the pheatmap package (v.1.0.12) in R.

### Statistical analysis

Growth performance data were subjected to analysis of variance via the general linear model procedure in SPSS Statistics software (v.23.0; IBM Corp., Armonk, NY, USA) (25). Intergroup comparisons of treatment means were performed using Tukey’s test, with all data expressed as mean values. Statistical significance was defined as *P* < 0.05. For the WFBG supplementation, dose–response effects were evaluated through orthogonal polynomial contrast analyses to assess linear and quadratic relationships.

## RESULTS

### Growth performance and intestinal development

During days 22–42, birds fed diets containing 5%, 10%, 20%, and 30% WFBG exhibited significantly lower ADG than the control group (0% WFBG, *P* < 0.05; Table 2). However, birds fed diets containing 5% and 20% WFBG had higher ADG than those fed diets with 10% WFBG (*P* < 0.05, Table 2). In terms of ADFI, birds fed diets containing 10% and 20% WFBG exhibited significantly lower ADFI than those fed diets with 0%, 5% and 30% WFBG (*P* < 0.05, Table 2). For FCR, birds fed diets containing 20% WFBG exhibited a significantly lower FCR than those fed diets with 5% and 30% WFBG from day 22 to 42 (*P* < 0.05, Table 2), while no significant differences in FCR were observed among the 0%, 10%, and 20% WFBG groups (*P* > 0.05, Table 2). Furthermore, the five experimental groups had similar survival rates throughout the trial (*P* > 0.05, Table 2).

**TABLE 2:** Effects of WFBG on growth performance in white-feathered broilers^*a*^

Items	CON group^*b*^	5% WFBG group^*b*^	10% WFBG group^*b*^	20% WFBG group^*b*^	30% WFBG group^*b*^	SEM^*c*^	*P* value^*d*^		
							Treatment	Linear	Quadratic
*n*	6	6	6	6	6				
BW (g)									
d 21	370.72^b^	404.78^a^	409.68^a^	397.04^a^	403.67^a^	3.39	<0.001	<0.001	0.002
d 28	655.06	650.42	649.11	655.14	648.74	3.59	0.967	0.734	0.872
d 35	831.25^ab^	831.49^ab^	835.33^a^	845.18^a^	788.13^b^	7.04	0.085	0.176	0.068
d 42	1,103.36^a^	1,089.94^ab^	1,037.09^b^	1,074.64^ab^	1,055.68^ab^	8.44	0.085	0.044	0.443
ADG (g)									
d 22-28	40.88^a^	35.09^b^	34.21^b^	37.30^b^	35.01^b^	0.66	0.003	0.005	0.018
d 29-35	25.17^a^	25.87^a^	26.60^a^	27.15^a^	19.91^b^	0.83	0.030	0.143	0.017
d 36-42	38.87^a^	36.92^a^	28.82^b^	32.78^ab^	38.22^a^	1.14	0.014	0.278	0.011
d 22-42	34.98^a^	32.63^b^	29.88^c^	32.41^b^	31.05^bc^	0.43	0.001	<0.001	0.035
ADFI (g)									
d 22-28	55.77	59.62	56.49	55.95	61.48	0.86	0.126	0.169	0.582
d 29-35	65.12^b^	67.27^ab^	70.42^ab^	64.81^b^	74.81^a^	1.33	0.081	0.064	0.471
d 36-42	98.37^a^	89.42^ab^	68.17^c^	78.03^bc^	98.13^a^	2.73	<0.001	0.128	<0.001
d 22-42	75.18^a^	73.63^a^	66.64^b^	65.94^b^	79.38^a^	1.24	<0.001	0.753	<0.001
FCR									
d 22-28	1.37^c^	1.70^ab^	1.66^ab^	1.52^bc^	1.76^a^	0.04	0.002	0.003	0.202
d 29-35	2.62^b^	2.63^b^	2.69^b^	2.45^b^	4.03^a^	0.16	0.003	0.013	0.011
d 36-42	2.53	2.43	2.41	2.45	2.61	0.06	0.836	0.812	0.269
d 22-42	2.15^bc^	2.26^b^	2.23^bc^	2.04^c^	2.56^a^	0.04	<0.001	0.011	0.031
Survival rate (%)	98.89	97.92	98.96	97.92	98.96	0.50	0.921	0.961	0.626

^*a*^ Means represent six replicates with 16 birds per cage (*n* = 6/group).

^*b*^ CON group: basal diet; 5% WFBG group: basal diet + 5% WFBG; 10% WFBG group: basal diet + 10% WFBG; 20% WFBG group: basal diet + 20% WFBG; 30% WFBG group: basal diet + 30% WFBG. ADFI, average daily feed intake; ADG, average daily gain; BW, body weight; FCR, feed conversion efficiency.

^*c*^ Standard error of the mean.

^*d*^ Values within a row with different superscripts differ significantly at *P* < 0.05. Treatment, treatment effect; linear, linear effect for treatment with WFBG levels of 0%, 5%, 10%, 20%, and 30%; quadratic, quadratic effect for treatment with WFBG levels of 0%, 5%, 10%, 20%, and 30%.

**Fig 1:**
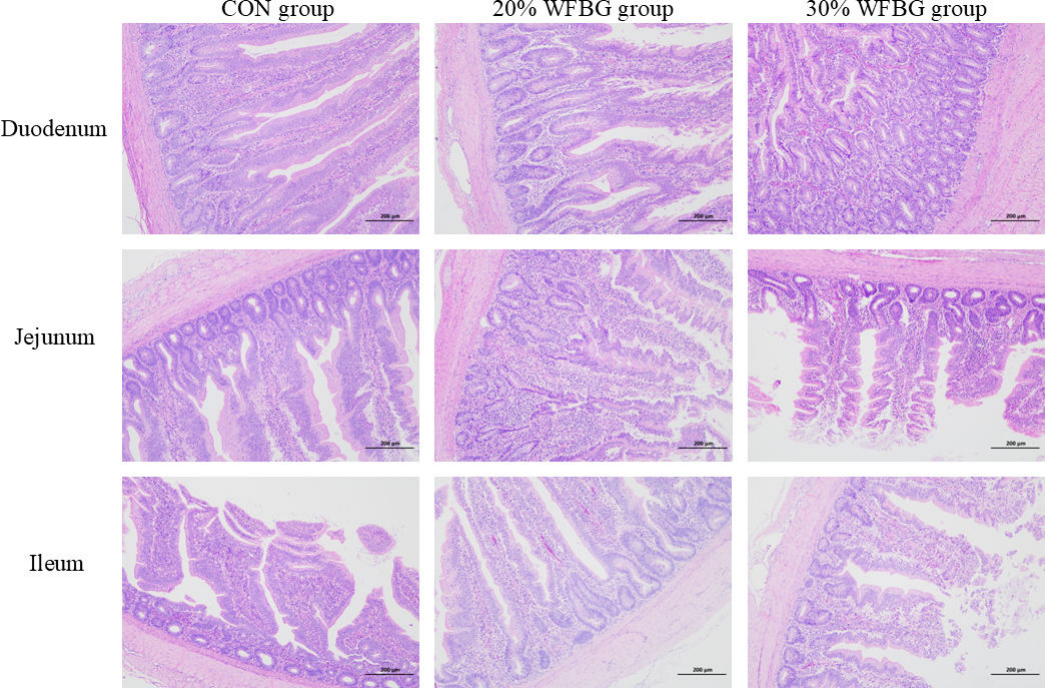
Effects of WFBG on the intestinal morphological structure of broilers. Control (CON) group (no WFBG addition), 20% WFBG group (diet with 20% WFBG inclusion), 30% WFBG group (diet with 30% WFBG inclusion). Rows correspond to different intestinal segments (duodenum, jejunum, and ileum), and columns correspond to different treatment groups (CON group, 20% WFBG group, and 30% WFBG group). Hematoxylin-eosin staining was used to visualize tissue structure; all images share a consistent scale bar of 200 µm.

Compared to the 30% WFBG group, the 20% WFBG group showed a significant increase in the duodenal crypt depth (CD) (*P* < 0.05, Table 3). Additionally, the duodenal villus height-to-crypt depth (VH/CD) ratio was significantly higher in both the control and 20% WFBG groups compared to the 30% WFBG group (*P* < 0.05, Table 3). The 20% WFBG group exhibited a significant increase in ileal VH compared to both the control and 30% WFBG groups (*P* < 0.05, Table 3). No significant differences were detected in the VH, CD, or VH/CD ratio of the jejunum nor in the CD or VH/CD ratio of the ileum (*P* > 0.05, Table 3). Intuitive morphological changes in these intestinal segments are further supported by HE-stained paraffin sections of broiler intestinal tissues from each group, which are presented in Fig. 1.

**TABLE 3:** Effects of dietary WFBG on the intestinal development in white-feathered broilers^*a*^

Items^*b*^	CON group^*c*^	20% WFBG group^*c*^	30% WFBG group^*c*^	SEM^*d*^	*P* value^*e*^
Duodenum					
VH (mm)	1.37^ab^	1.64^a^	1.25^b^	0.07	0.039
CD (mm)	0.31^b^	0.34^ab^	0.41^a^	0.02	0.084
VH/CD	4.55^a^	4.91^a^	3.08^b^	0.25	0.002
Jejunum					
VH (mm)	0.78	0.85	0.65	0.04	0.193
CD (mm)	0.29	0.42	0.26	0.04	0.159
VH/CD	2.85	2.30	2.52	0.15	0.324
Ileum					
VH (mm)	0.56^b^	0.70^a^	0.56^b^	0.03	0.052
CD (mm)	0.20	0.23	0.20	0.01	0.326
VH/CD	3.06	3.08	2.86	0.16	0.849

^*a*^ Means represent six replicates with 16 birds per cage (*n* = 6/group).

^*b*^ CD, crypt depth; VH, villus height.

^*c*^ CON group: basal diet; 20% WFBG group: basal diet + 20% WFBG; 30% WFBG group: basal diet + 30% WFBG.

^*d*^ Pooled standard error of the mean.

^*e*^ Values within a row with different superscripts differ significantly at *P* < 0.05.

### WFBG exhibited alterations of gut microbiota composition and functionality

**Fig 2:**
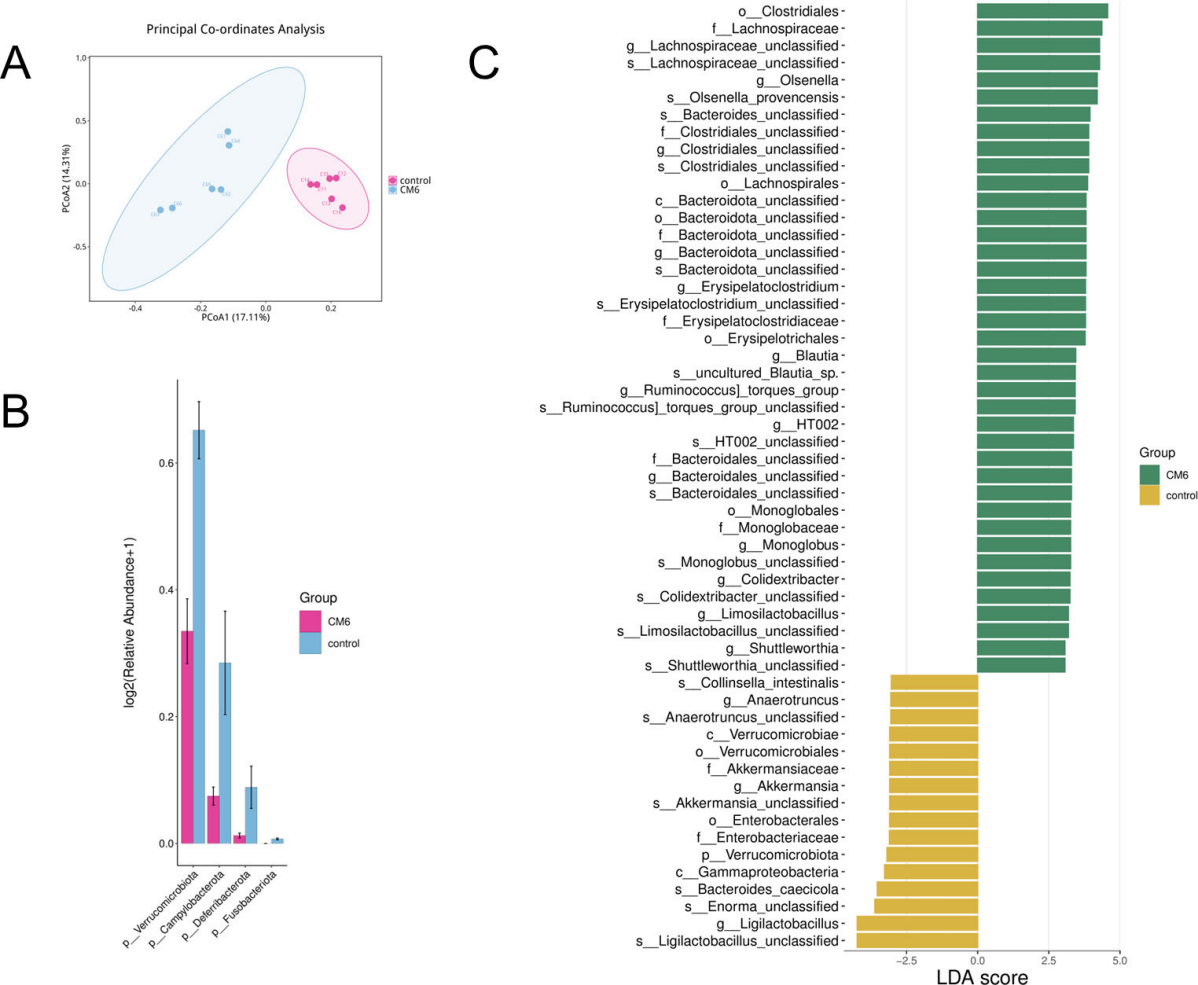
Taxonomic and functional profiles of gut microbiota in WFBG-supplemented broilers vs normal birds (control group). (**A**). Principal coordinate analysis (PCoA) plot showing species-level beta diversity based on the Aitchison distance metric, clearly distinguishing the control group (red) from the WFBG-supplemented group (blue). (**B**). Compositional bar plots illustrating the taxonomic profiles of gut microbiota at the phylum, family, and order levels. (**C**). MaAsLin2-based multivariate differential abundance analysis of bacterial genera, comparing the control group with the WFBG-supplemented group.

Previous studies have demonstrated that WFBG modulates the composition and functional activity of pullet gut microbiota, yet its impact on broiler intestinal microbiota has not been explored hitherto. To fill this research void, cecal specimens were obtained from the control and 20% WFBG groups and subjected to metagenomic analysis (*n* = 6). No significant group-wise differences in bacterial alpha diversity (genus-level, Shannon index) were observed via 16S rRNA sequencing analysis between the two groups (Fig. S1A). Notably, species-level beta-diversity analysis via Aitchison distance-based principal coordinate analysis indicated significant dissimilarities in bacterial community structures between the control and WFBG groups (*P* = 0.002 and *R*^2^ = 0.21, permutational multivariate analysis of variance) (Fig. 2A). 16S rRNA sequencing analysis indicated significant differences in the relative abundances of *Verrucomicrobiota*, *Campylobacterota*, *Deferribacterota*, and *Fusobacteriota* at the phylum level in broiler gut microbiota between the control and WFBG groups (Fig. 2B). Specifically, these taxa were significantly downregulated in the WFBG group compared to the control group. At the genus level, the most abundant taxa were *Ligilactobacillus*, *Olsenella*, *Erysipelatoclostridium*, and *Blautia*, which showed significant differences between the control and WFBG groups (Fig. S1B). Notably, *Ligilactobacillus* exhibited a significant decrease in the 20% WFBG group compared to the control group, whereas *Olsenella*, *Erysipelatoclostridium*, and *Blautia* were significantly upregulated. A linear regression model revealed significant differences between the control and WFBG groups (Fig. 2C; Table S1). The main change at the order level was an increase in *Clostridiales* abundance in broilers fed diets with 20% WFBG (Fig. S1C). Compared to broilers in the control group, broilers fed diets containing WFBG had higher relative abundances of *Olsenella_provencensis* and *uncultured_Blautia_sp*. (Fig. S1D) at the species level. In the control group, enriched species included *Bacteroides_caecicola* and *Collinsella_intestinalis* at the species level (Fig. S1D).

### Serum untargeted metabolomics profiles of birds fed diets with WFBG

**Fig 3:**
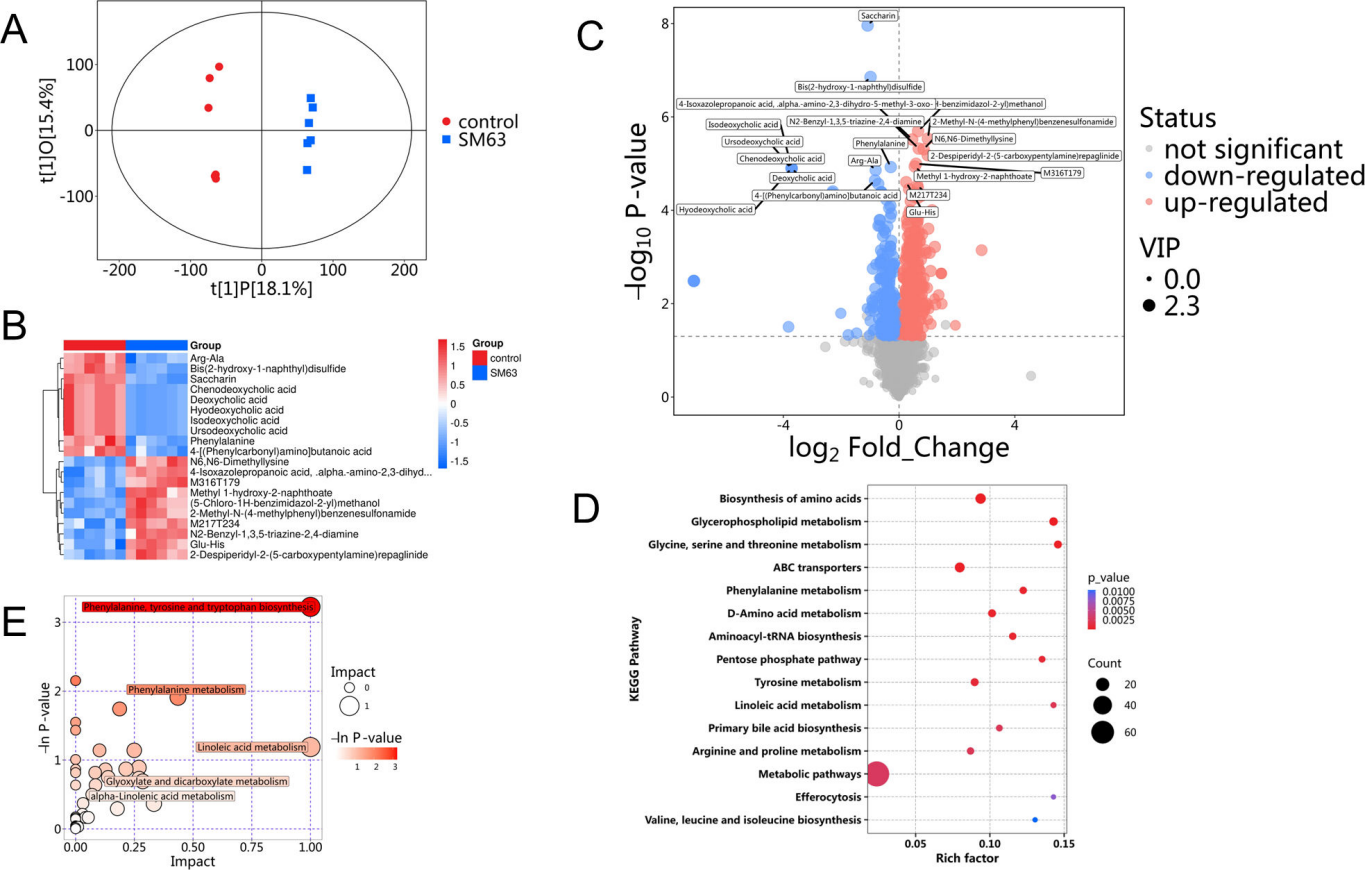
Serum metabolome analysis in WFBG-supplemented broilers vs normal birds (control). (**A**) OPLS-DA analysis of metabolite profile alterations. (**B**) Cluster heat map (green, lower abundance; red, higher abundance). (**C**) Volcano plot of DEMs between control and 20% WFBG groups (*t*-test *P* < 0.05, fold change >1.0 or <1.0). Gray, no change; red, upregulated; green, downregulated. (**D**) KEGG pathway analysis. Dot size: number of DEMs enriched; color: *P* value (redder = smaller *P* value = more significant). (**E**) Metabolic pathway analysis. Each bubble represents a pathway. Horizontal axis/bubble size: topological influence factor, vertical axis/color: -ln (enrichment *P* value).

**Fig 4:**
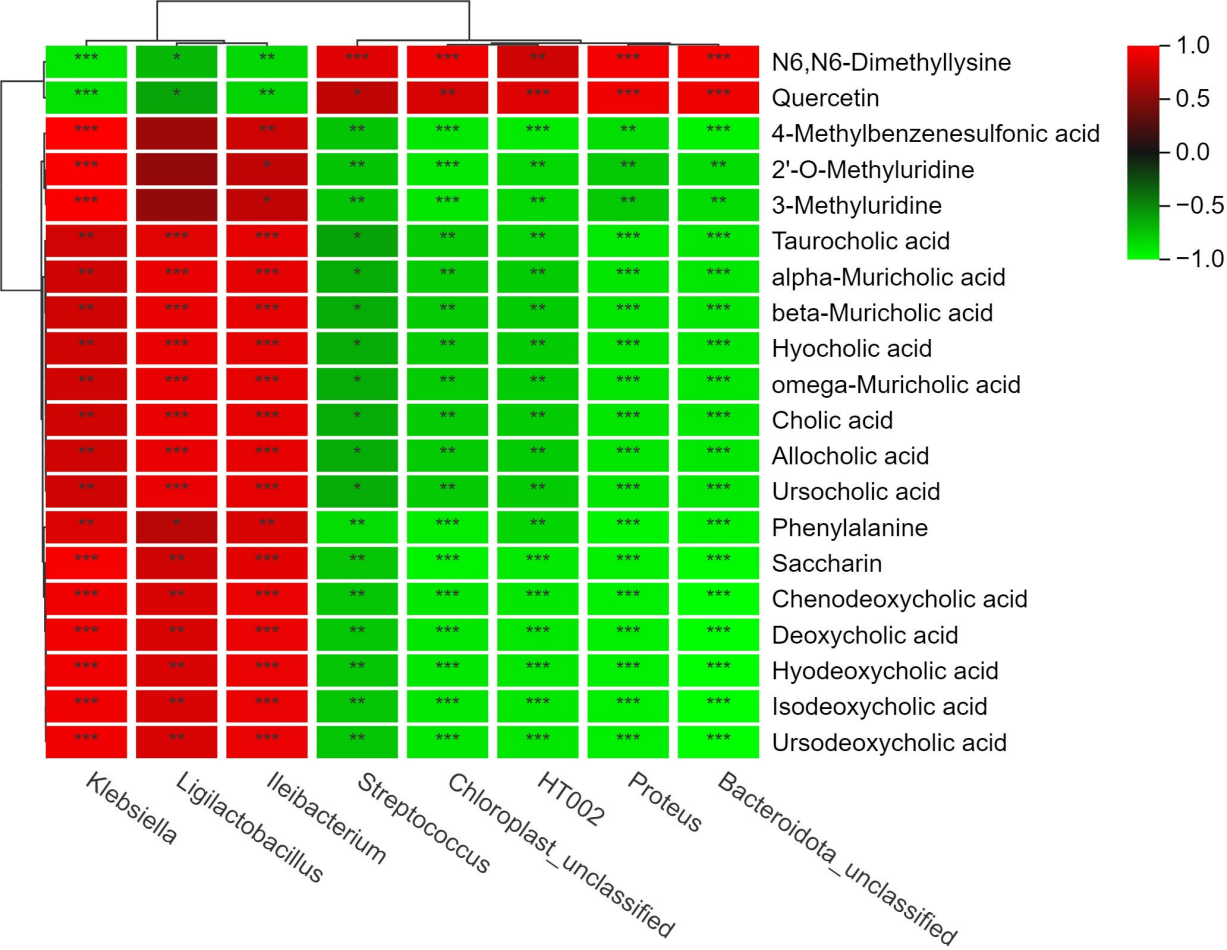
WFBG-associated networks based on shared pathways between serum metabolome and cecal microbiota (Spearman’s rank correlation analysis, *r* > 0.8, *P* < 0.05). The number of asterisks (*) indicates the degree of correlation (*P < 0.05, **P < 0.01, ***P < 0.001). Red and green represent positive and negative correlations, respectively.

To investigate the metabolic alterations in broilers fed high-dose WFBG, we performed non-targeted metabolomic analysis. Orthogonal partial least squares discriminant analysis (OPLS-DA) was applied to visualize the clustering patterns of metabolic profiles and screen for DEMs across groups. Serum metabolomic profiling showed that broilers fed a 20% WFBG diet exhibited significant shifts in their metabolic signatures compared to the control group (Fig. 3A). The serum OPLS-DA score plots clearly displayed distinct clustering patterns between the control and WFBG groups (R2X = 0.335, R2Y = 0.996, *Q*2 = 0.771) (Fig. 3A), demonstrating the OPLS-DA models exhibited strong robustness without overfitting. The screening criteria for identifying DEMs in the serum of broilers from the 20% WFBG and control groups were set as a VIP score of >1.0 and a *P* value of <0.05. Heat maps were used to visualize the relative abundance of serum DEMs between the two groups (Fig. 3B). A total of 546 DEMs were identified in the serum of the 20% WFBG group, including 323 upregulated and 223 downregulated DEMs compared to the control group (Fig. 3C). Among these DEMs, 3-hydroxybutyric acid (β-OHB), glyceric acid, and ribitol related to energy metabolism were upregulated. In amino acid metabolism, N6,N6-dimethyllysine, acetylglycine, and n6-methyllysine were upregulated, while L-4-hydroxyglutamate_semialdehyde, 1-(3,5-dimethyl-1H-pyrazol-4-yl) ethanamine, and D,L-threo-3-hydroxyaspartic acid were downregulated. In lipid metabolism, N-tetracosenoyl-4-sphingenine and PC[18:4(6Z,9Z,12Z,15Z)/22:6 (4Z,7Z,10Z,13Z,16Z,19Z)] were downregulated. For antioxidant regulation, quercetin and cysteine-glutathione disulfide were upregulated, while macluraxanthone showed downregulation. 9-(Furan-3-yl)−5-hydroxy-5a,9a,14,14-tetramethyl-5a,9,9a,10,11,11a-hexahydro-3a,11b-(methanooxymethano)indeno[5,4-f]oxireno[d]isochromene-3,4,7(3aH,5H,6aH)-trione also showed downregulation, but its antioxidant function needed to be further validated by combining structural similarity. In other metabolic pathways, quinic acid and 3-amino-2-piperidone were upregulated, while papaverine, 4-formylaminoantipyrine, and sulfoxone showed downregulation (Fig. 4). The top 10 DEMs in the serum between the 20% WFBG and control groups are presented in Table 4. Extended details can be found in Table S2.

**TABLE 4:** Identification of top 10 DEMs in the serum between the 20% WFBG and control groups^*a*^

No.	Metabolites	*m*/*z*	RT (s)	VIP	FC	Mode	*P* value
1	Cysteine-glutathione disulfide	427.10	268.4	2.01	1.50	Up	0.0006
2	Quercetin	301.04	13.5	2.15	1.27	Up	3.53E-05
3	Quinic acid	191.06	196.2	2.01	1.66	Up	0.0005
4	N6, N6-dimethyllysine	175.14	306.6	2.24	1.80	Up	4.94E-06
5	N6-methyllysine	161.13	303.8	1.98	1.62	Up	0.0039
6	Ribitol	151.06	104.5	1.96	1.51	Up	0.0053
7	Acetylglycine	116.04	160.9	2.11	1.50	Up	0.0007
8	3-amino-2-piperidone	115.09	103.8	2.01	1.34	Up	0.0029
9	Glyceric acid	105.02	172.9	1.99	1.27	Up	0.0035
10	3-Hydroxybutyric acid	103.04	125.8	2.13	1.56	Up	0.0018
11	Allocholic acid	407.28	128.5	2.26	0.01	Down	0.0033
12	Ursocholic acid	407.28	128.5	2.26	0.01	Down	0.0033
13	Cholic acid	407.28	128.5	2.26	0.01	Down	0.0033
14	Omega-muricholic acid	407.28	128.5	2.26	0.01	Down	0.0033
15	Hyocholic acid	407.28	128.5	2.26	0.01	Down	0.0033
16	Beta-muricholic acid	407.28	128.5	2.26	0.01	Down	0.0033
17	Alpha-muricholic acid	407.28	128.5	2.26	0.01	Down	0.0033
18	Isodeoxycholic acid	391.28	50.2	2.27	0.08	Down	1.26E-05
19	Ursodeoxycholic acid	391.28	50.2	2.27	0.08	Down	1.26E-05
20	Hyodeoxycholic acid	391.28	50.2	2.27	0.08	Down	1.26E-05

^*a*^ DEM, different expressed metabolite; FC, fold change, 20% WFBG group vs control group; *m*/*z*, mass-to-charge ratio; RT, retention time; VIP, variable importance in the projection; WFBG, wet fermented brewer's grain.

A total of 2,071 serum metabolites identified in positive and negative ion modes were subjected to KEGG pathway analysis, among which 546 were DEMs (Table S3). The functional impacts of enriched DEMs on metabolic pathways were analyzed using the MetaboAnalyst (Fig. 3D). A total of 74 serum metabolic pathways were significantly altered in the 20% WFBG group (*P* < 0.05), including metabolic pathways (73), biosynthesis of amino acids (12), ABC transporters (11), glycerophospholipid metabolism (8), glycine, serine, and threonine metabolism (7), tyrosine metabolism (7), D-amino acid metabolism (7), carbon metabolism (6), 2-oxocarboxylic acid metabolism (6), and arginine and proline metabolism (6). Notably, the top 5 pathway impact values in the serum for phenylalanine, tyrosine and tryptophan biosynthesis, linoleic acid metabolism, phenylalanine metabolism, alpha-linolenic acid metabolism, and glyoxylate and dicarboxylate metabolism were 1.000, 1.000, 0.436, 0.333, and 0.286, respectively (Fig. 2E; Table 5). Thus, we further explored metabolic changes in these pathways among broilers fed diets with 20% WFBG. Key findings showed upregulation of L-tyrosine, linoleic acid, alpha-linolenic acid, cis-aconitic acid, and glyceric acid, alongside downregulation of L-phenylalanine.

**TABLE 5:** Significantly altered metabolic pathways and matched metabolites in the serum of broilers fed 20% WFBG (*P* < 0.05)

Metabolic pathways	Impact value	*P* value	Matched metabolites
Phenylalanine, tyrosine, and tryptophan biosynthesis	1.000	0.040	L-phenylalanine and L-tyrosine
Linoleic acid metabolism	1.000	0.305	Linoleic acid
Phenylalanine metabolism	0.436	0.148	L-phenylalanine and L-tyrosine
Alpha-linolenic acid metabolism	0.333	0.694	Alpha-linolenic acid
Glyoxylate and dicarboxylate metabolism	0.286	0.501	cis-Aconitic acid and glyceric acid

Detailed pathway analysis results are presented in the Table S4.

### Serum metabolite–gut microbiota correlation analysis

An integrative analysis combining metagenomics and metabolomics was subsequently conducted to identify relevant associations (Table S4). The Spearman rank correlation coefficient was applied to quantify associations between continuous variables. Significant correlations were visualized as a heat map, where each row/column represented a feature (bacterial taxa or metabolites), and color gradients denoted pairwise correlations (warm colors for positive and cool colors for negative). Statistical significance was indicated by asterisks (*P < 0.05, **P < 0.01, ***P < 0.001). By contrasting the 20% WFBG and control groups, microbial-metabolite co-occurrence networks were constructed for broilers (Fig. 3).

The gut bacterial genera *Streptococcus*, *Proteus*, *HT002*, unclassified chloroplast, and unclassified *Bacteroidetes* showed positive associations with two DEMs (N6,N6-dimethyllysine and quercetin). Conversely, these genera showed negative correlations with 18 DEMs, including 4-methylbenzenesulfonic acid, saccharin, isodeoxycholic acid, ursodeoxycholic acid, and hyodeoxycholic acid. Similarly, *Klebsiella*, *Ligilactobacillus*, and *Ileibacterium* were positively linked to the same two DEMs and negatively correlated with the identical set of 18 DEMs (e.g., 4-methylbenzenesulfonic acid, saccharin, and the deoxycholic acid derivatives). Collectively, these findings illuminate distinct microbe–metabolite associations between the control and WFBG groups, underscoring their potential role in metabolic differentiation.

## DISCUSSION

Previous studies have evidenced alterations in growth performance and hepatic metabolites of pullets (26) or broilers (23) fed WFBG-supplemented diets. However, the metabolic and metagenomic profiles of broiler individuals remain poorly characterized. Thus, clinical trials serve as valuable tools to dissect the potential impact of WFBG-supplemented diets on broilers. In this study, we employed 16S rRNA gene sequencing and untargeted metabolomics to identify bacterial and metabolic markers associated with WFBG exposure, revealing its influence on gut microbiota composition and growth performance.

### Growth performance and intestinal morphology

Previous studies have reported conflicting effects of DBG and WFBG on broiler growth performance. While 2%–10% DBG showed no impact (27), 2%–12% DBG reduced ADG and increased FCR (3), and 9% DBG decreased both ADG and ADFI (28). Ten percent dry FBG promoted a 12% BW increase, but 15% reduced ADFI (2). Wet fermentation may enhance WFBG palatability to increase feed intake, as seen in geese (29) and pigs (30). The ADG enhancement is likely attributed to increased feed consumption induced by wet feeding and fermentation. Intestinal development quality critically affects animal growth. Crypt-villus stem cell renewal influences villus morphology and absorptive function. Higher VH/CD ratio indicates better digestive efficiency. Previous studies showed 2%–10% DBG did not promote intestinal development in broilers (3, 27). Notably, our prior research showed 20% WFBG reduced pullet ADFI without affecting weight gain and promoted duodenal development in pullets (26) and broilers (23). In this study, broilers fed WFBG diets showed lower ADG, with 10% and 20% WFBG reducing ADFI. The 20% group had a lower FCR than 5%/30% groups, matching the control and contradicting prior reports. Reduced growth may stem from limited crude fiber digestibility, yet 20% WFBG improved FCR, suggesting feed cost savings. Excessive WFBG (30%) impaired duodenal villus morphology—likely due to increased intestinal metabolic burden—while sparing jejunal/ileal development; these effects, potentially linked to dietary fiber dilution or gut microbiota shifts, also lay the groundwork for understanding FCR variations across groups. By contrast, the improved FCR in the 20% WFBG group confirms the speculation that wet fermentation enhances feed conversion efficiency: the process may degrade antinutritional factors (e.g., crude fiber), and wet feeding further boosts intestinal nutrient absorption, consistent with prior findings in geese (29) and pigs (30) that wet fermentation improves palatability and digestibility. Conversely, the damaged duodenal villus morphology in the 30% group clarifies why its FCR rebounded: excessively high inclusion inhibits intestinal function via heightened metabolic burden or fiber dilution, directly counteracting the efficiency gains seen in the 20% group.

Notably, the protective effect of 20% WFBG on jejunal and ileal development in this study echoes the previous findings that “20% WFBG promotes duodenal development in pullets and broilers” (23, 26). This further indicates that the regulation of WFBG on intestinal development exhibits “intestinal segment-specificity,” and its interaction mechanism with intestinal microbiota still requires subsequent verification. As a low-cost by-product of the brewing industry, the core application value of WFBG in poultry production lies not in “simply increasing the inclusion level” but in optimizing costs through “reducing conventional feed consumption” and “improving feed economic efficiency.” Safety is a prerequisite for economic application: this experiment confirms 20% WFBG is safe for broilers. Its economic advantages surpass 5% group: lower FCR reduces feed consumption per unit gain (direct benefit); 20% inclusion maximizes replacing high-cost ingredients (e.g., soybean meal, 4,000 yuan/ton) with low-cost WFBG (<1,000 yuan/ton, indirect benefit). The 5% group exhibits a higher FCR and lacks economic merits, while the 30% group poses health risks. In summary, the economic value of WFBG must be based on the “safe inclusion level” and centered on “improved FCR + high substitution rate.” The 20% inclusion level just meets these two conditions, providing dual support of “technical feasibility + economic rationality” for its promotion in broiler production. However, the 5% and 30% groups are not suitable as preferred options due to “insufficient economic benefits” or “increased health risks.”

### WFBG gastrointestinal exposure induced metabolic disturbances

Upon supplementation with 20% WFBG, 42-day-old broilers exhibited significant alterations in cecal microbiota composition, as confirmed by beta-diversity analysis. The 20% WFBG group showed enrichment of the *Clostridiales* order, with notable increases in *Olsenella* and uncultured *Blautia* spp. Conversely, *Bacteroides caecicola* and *Collinsella intestinalis*—species typically abundant in healthy guts—were less prevalent. *Clostridiales* comprises both beneficial taxa (e.g., SCFA producers) and potential pathogens. Their enrichment may reflect altered gut fermentation or microenvironmental shifts. *Olsenella* aids carbohydrate metabolism and fiber degradation (31), while *Blautia* are renowned butyrate producers (32). The “uncultured” *Blautia* strains highlight functional roles requiring validation. *Bacteroides caecicola* facilitates fiber digestion (33), and *Collinsella intestinalis* supports intestinal homeostasis and immune regulation (34). Increased *Clostridiales* (e.g., *Blautia*) may enhance butyrate production to promote intestinal epithelial health, yet overgrowth of pathogenic strains could induce inflammation. Reduced *Bacteroides* and *Collinsella* may impair fiber digestion and weaken gut barriers, increasing pathogen susceptibility. Enhanced duodenal development likely stems from butyrate-mediated villus morphology improvement, as *Clostridiales*-derived SCFAs promote epithelial cell proliferation (35). These findings indicate dual effects of 20% WFBG: *Clostridiales*-driven butyrate production may support epithelial health, while depletion of *Bacteroides* and *Collinsella* disrupts fiber digestion and barrier function. Optimal dosage and probiotic synergies warrant investigation to resolve microbial dysbiosis and morphological improvements.

Regarding microbiota shifts, *Ligilactobacillus* abundance significantly decreased in the 20% WFBG group vs controls, whereas *Olsenella*, *Erysipelatoclostridium*, and *Blautia* levels were elevated. As a gut commensal, *Ligilactobacillus* forms a biofilm barrier to inhibit *Salmonella* colonization, ferments carbohydrates to produce lactic acid (suppressing *Escherichia coli*), and stimulates mucosal immunity. It also decomposes fiber into SCFAs (e.g., butyrate) to energize enterocytes and enhance mineral absorption (36). *Olsenella* (Actinobacteria) degrades arabinan to generate acetic acid, supports immune homeostasis via T-cell regulation, and proliferates under high-fiber diets in pigs (37). *Erysipelatoclostridium* (Firmicutes) is a major butyrate producer, preserving gut barriers and inhibiting NF-κB inflammation, with ruminant abundance correlating with cellulose digestion (38). *Blautia* (Firmicutes) promotes epithelial proliferation and bile acid metabolism, with probiotic intervention boosting its abundance in broilers (39). Human/animal studies link *Blautia* to fiber intake (positively) and constipation/enteritis (negatively) (40). Collectively, *Blautia* is the most fiber sensitive, *Ligilactobacillus* responds to probiotics, and *Olsenella* mirrors host metabolic status. Non-targeted metabolomic analysis indicated that 20% WFBG supplementation activated the phenylalanine, tyrosine, and tryptophan biosynthesis pathways, as well as the alpha-linolenic acid metabolism pathway in broilers, which might explain the improved intestinal development and feed conversion efficiency. Whether long-term WFBG affects meat quality and immune organ development requires validation via meat quality assays and further mechanistic studies. These findings highlight 20% WFBG remodels broiler gut microbiota to potentially enhance gut health, with some shifts linked to SCFA production, warranting further exploration. To clarify this link and deepen characterization of WFBG-induced microbiota changes, we will investigate SCFA profiles (acetate, propionate, and butyrate) in subsequent studies and integrate these metabolites with current microbiota data to fully elucidate WFBG’s gut health mechanism.

### Gut microbiota–serum metabolite integrated analysis

The integrated analysis of non-targeted metabolomics and cecal microbiota revealed that in 20% WFBG-treated broilers, two DEMs—N6,N6-dimethyllysine and quercetin—were significantly up-regulated, engaging in amino acid modification/epigenetic regulation and antioxidant/anti-inflammatory processes, respectively. N6,N6-Dimethyllysine mediates amino acid modification and epigenetic regulation via histone methylation, and quercetin exerts antioxidant and anti-inflammatory effects through scavenging free radicals and inhibiting NF-κB signaling. Bacterial taxa including *Streptococcus* and *Proteus* showed positive correlations with these DEMs while negatively associating with 18 DEMs (e.g., 4-methylbenzenesulfonic acid and deoxycholic acid derivatives). This study characterized the key metabolite–microbiota interaction network, indicating that N6,N6-dimethyllysine and quercetin may act as hub molecules for microbial modulation of host metabolism, providing mechanistic insights into WFBG-mediated metabolic regulation in broilers.

### Conclusions

In summary, 20% WFBG-treated broilers showed lower ADG than that in the control group but significantly higher ADG than that in the 10% and 30% groups. FCR was improved, with better duodenal and ileal intestinal morphology. The analysis of gut microbiota structure showed a significant decrease in the abundance of *Ligullaceae*, while genera such as *Olsenella*, *Erysipelattoclostridium*, and *Blautia* were significantly enriched. A total of 546 DEMs were detected in the serum metabolome, significantly enriched in pathways such as phenylalanine, tyrosine, tryptophan biosynthesis, and alpha-linolenic acid metabolism. By integrating 16S rRNA sequencing with non-targeted metabolomic analysis, it was found that bacterial genera such as *Streptococcus* and *Proteus* were positively correlated with N6, N6-dimethyllysine, and quercetin, and negatively correlated with 18 DEMs (e.g., 4-methylbenzenesulfonic acid and deoxycholic acid derivatives). Research has confirmed that WFBG can be used as a non-traditional feed ingredient for developing low-cost value-added animal feed. A 20% maximum inclusion rate is recommended to prevent impairment of growth performance. This study provides a theoretical basis and technical support for the precise application of WFBG in low-carbon broiler production.

## Data Availability

The data set compiled for this study is provided as supplemental material accompanying this article. The metabolomics data have been deposited to the MetaboLights repository (https://www.ebi.ac.uk/metabolights/) with the study identifier MTBLS13352.

## SUPPLEMENTAL MATERIAL

Text S1 aem.01890-25-s0001.docx Detailed parameters for the metabolomics analysis.

Fig. S1 aem.01890-25-s0002.docx Rarefaction curves of alpha diversity and column chart for gut microbiota community differential analysis.

Table S1 aem.01890-25-s0003.xlsx Gut microbiota.

Table S2 aem.01890-25-s0004.xlsx Detailed parameters of identified significant metabolites.

Table S3 aem.01890-25-s0005.xlsx Serum metabolite details.

Table S4 aem.01890-25-s0006.xlsx KEGG pathway details.

ASM does not own the copyrights to Supplemental Material that may be linked to, or accessed through, an article. The authors have granted ASM a non-exclusive, world-wide license to publish the Supplemental Material files. Please contact the corresponding author directly for reuse.
