# Supplementary material for: Metabolomic and metagenomic insights into WFBG-mediated regulation of gut microbiota and metabolism in broilers
Source: Appl Environ Microbiol. 2025 Dec 8;92(1):e01890-25. doi: 10.1128/aem.01890-25 (PMC12838195; doi:10.1128/aem.01890-25)
Supplement: Text S1 — Detailed parameters for the metabolomics analysis. [file aem.01890-25-s0001.docx]

**Metabolomics data acquisition**

The LC gradient elution program was set at a flow rate of 0.3 ml/min as follows: 10%-25% solvent B from 0 to 4 min; 25%-55% solvent B from 4 to 7 min; 55%-100% solvent B from 7 to 28 min; 100% solvent B held for 28-29 min; and 100%-10% solvent B from 29 to 30 min. The column temperature was maintained at 30 °C.

The ESI source parameters for the mass spectrometer were configured as follows: sheath gas flow rate at 50 Arb, Aux gas flow rate at 15 Arb, capillary temperature maintained at 320 °C, full MS resolution set to 60,000, MS/MS resolution at 15,000, collision energy with stepped normalized collision energy (SNCE) of 20/30/40, and spray voltage of 3.8 kV in positive ion mode or -3.4 kV in negative ion mode.

**Multivariate statistical analysis**

The raw data were transformed into mzXML format via ProteoWizard and processed using a custom in-house program. Developed with R and built upon XCMS, this program enabled feature detection, extraction, alignment, and integration. For metabolite identification, the R package was utilized alongside BiotreeDB (V3.0).

In this research, X features were detected, and after denoising via relative standard deviation (RSD), X metabolites remained. Missing values were imputed with half of the minimum detected value, and data normalization was performed using an internal standard method. The final dataset, including feature numbers, sample names, and normalized feature areas, was imported into SIMCA 18.0.1 (Sartorius Stedim Data Analytics AB, Umea, Sweden) for multivariate analysis. To mitigate noise and high-variance effects, data were scaled and log-transformed prior to unsupervised principal component analysis (PCA), which visualized sample distribution and grouping. Potential outliers were identified using a 95% confidence interval threshold in PCA score plots.

Supervised orthogonal projections to latent structures discriminate analysis (OPLS-DA) was employed to visualize group separation and identify significantly altered metabolites. A 7-fold cross-validation was then carried out to compute the R² and Q² values. R² reflects the proportion of variable variance explained by the model, while Q² indicates the predictive capacity of the model. To validate the robustness and predictive performance of the OPLS-DA model, 200 permutation tests were performed to derive the intercept values of R² and Q². Specifically, the Q² intercept value serves as an indicator of model robustness, overfitting risk, and reliability, with lower values denoting better model quality.

Additionally, the variable importance in projection (VIP) scores for the first principal component of the OPLS-DA model were extracted. VIP values quantify the relative contribution of each metabolite to group discrimination. Metabolites with VIP > 1 and *P* < 0.05 (determined by Student's *t*-test) were designated as significantly altered. Pathway enrichment analysis was performed using public databases, including KEGG (<http://www.genome.jp/kegg/>) and MetaboAnalyst (<http://www.metaboanalyst.ca/>), to identify perturbed metabolic pathways.
