# Supplementary material for: Metabolomic and metagenomic insights into WFBG-mediated regulation of gut microbiota and metabolism in broilers
Source: Appl Environ Microbiol. 2025 Dec 8;92(1):e01890-25. doi: 10.1128/aem.01890-25 (PMC12838195; doi:10.1128/aem.01890-25)
Supplement: Fig. S1 — Rarefaction curves of alpha diversity and column chart for gut microbiota community differential analysis. [file aem.01890-25-s0002.docx]

**Figure S1. Rarefaction curves of alpha diversity and column chart for gut microbiota community differential analysis.** A. Alpha diversity rarefaction curves (Shannon index). The rarefaction curves of samples clustered at 95% sequences identity. B-D. Bacterial genus (B), order (C) and species (D) distributions analyzed by V4 amplicon sequencing of top 30 OTUs. For each dosage of WFBG (Control-0%, CM6-20% WFBG).


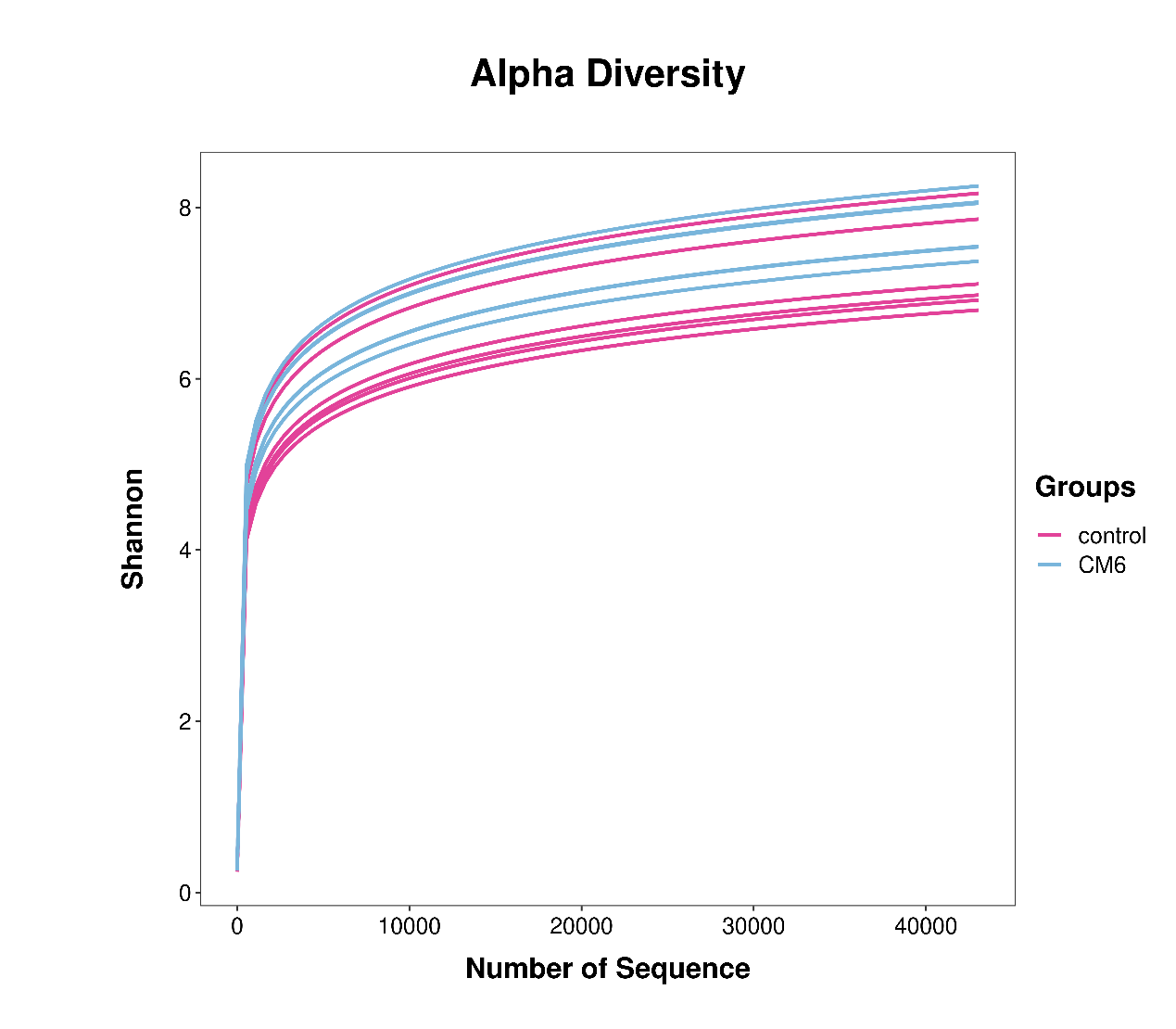


A


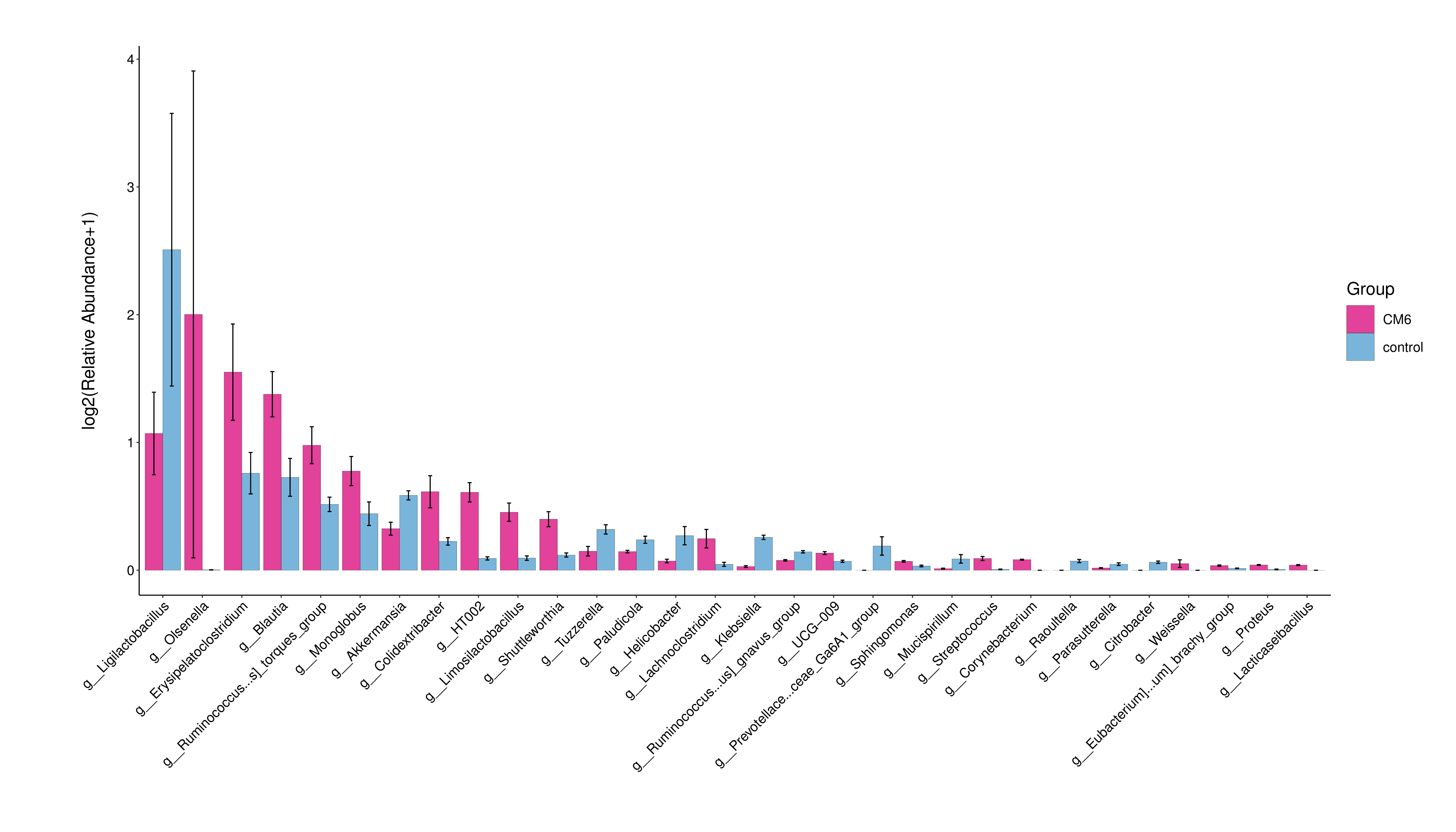


B


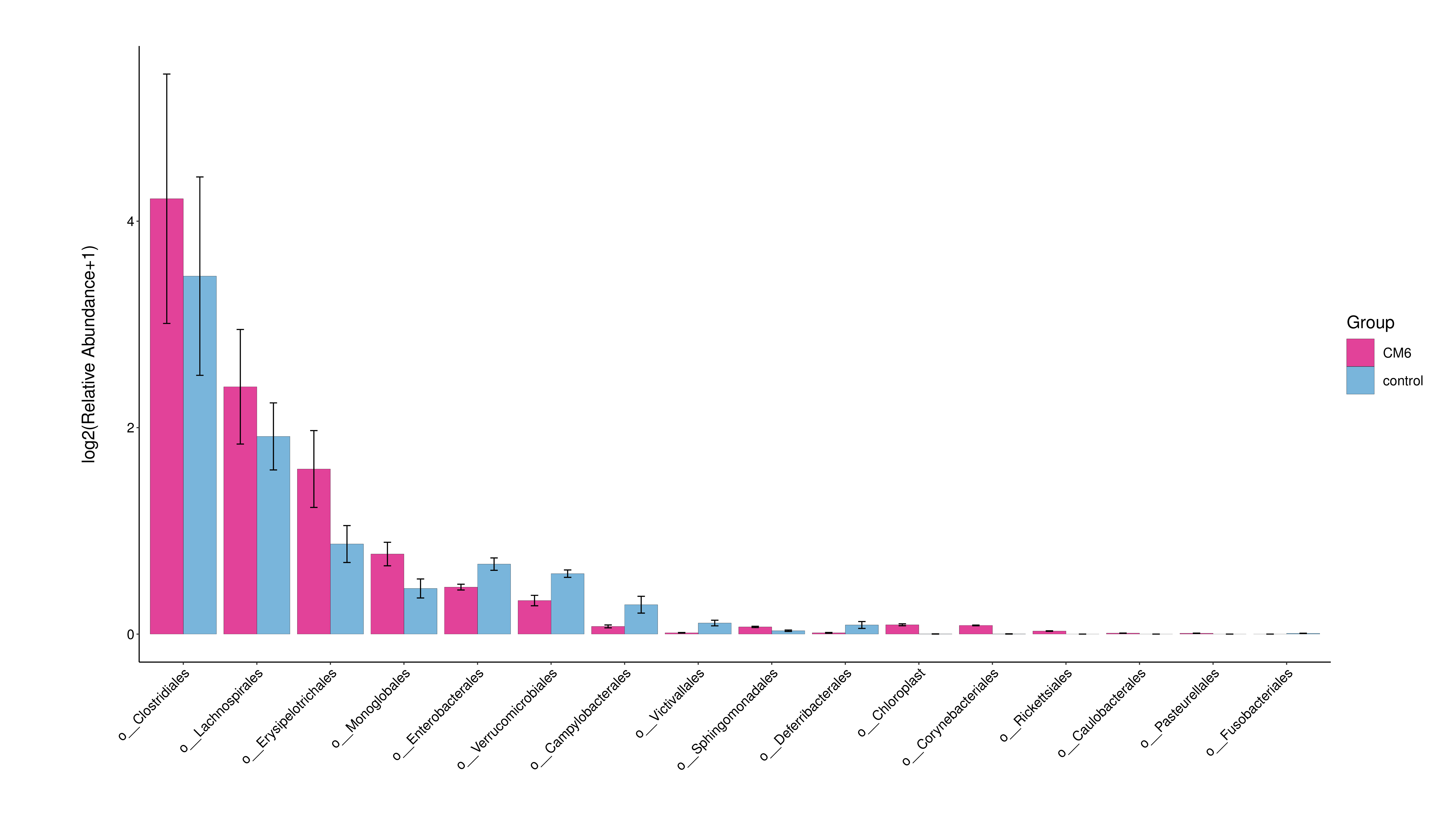


C


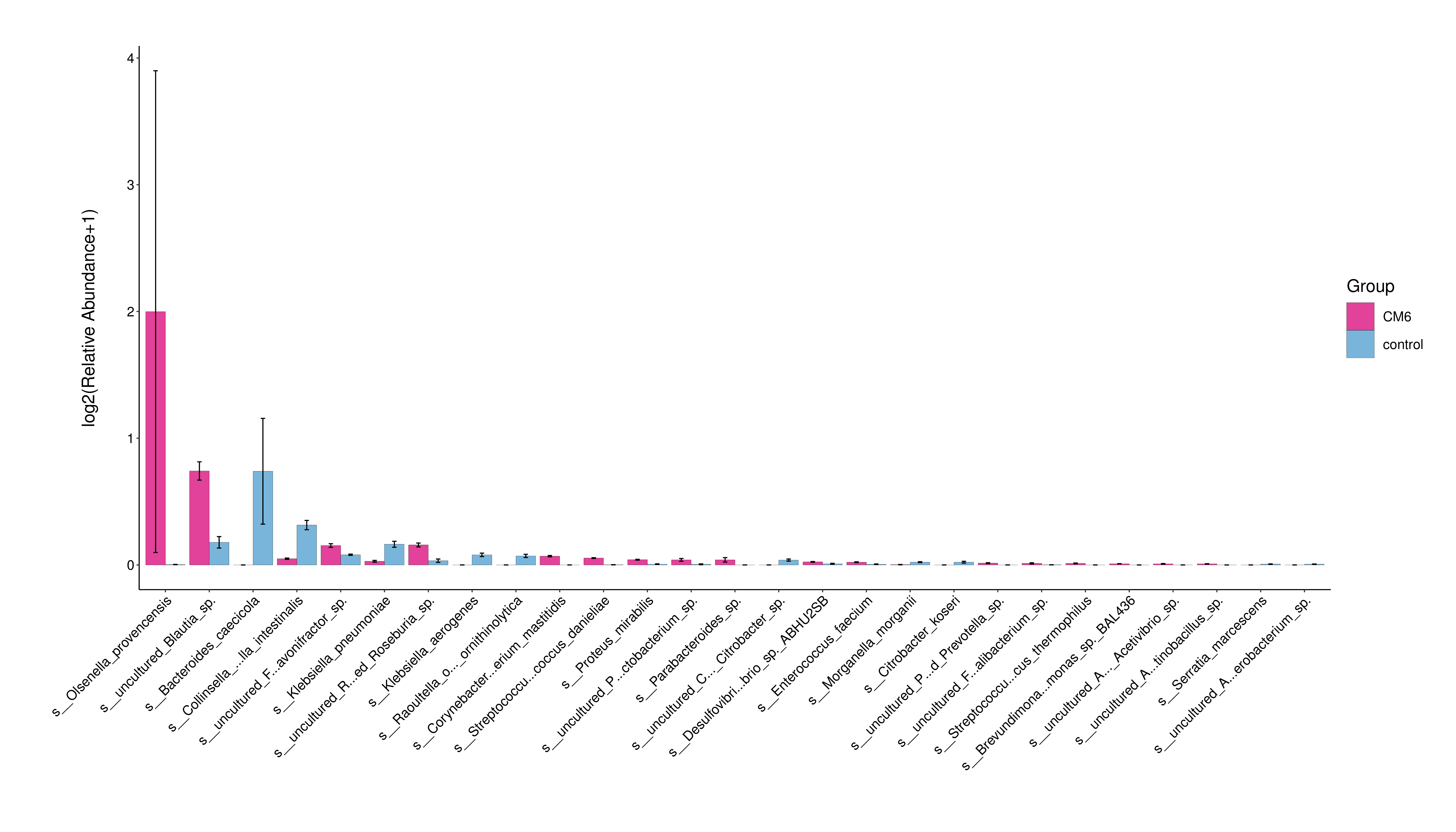


D
